# Performance of Seven-Gene Panel Testing for Risk Stratification of Thyroid Nodules with Indeterminate Cytology Results

**DOI:** 10.3390/ijms27114990

**Published:** 2026-05-30

**Authors:** Ann-Kristin Jochum, Frida Renström, Barbara Bischofberger-Baumann, Izadora Demmer, René Schönegg, Michael Brändle, Stefan Bilz, Wolfram Jochum

**Affiliations:** 1 Institute of Pathology, HOCH Health Ostschweiz, 9007 St. Gallen, Switzerland; ann-kristin.jochum@h-och.ch (A.-K.J.); izadora.demmerbuchs@h-och.ch (I.D.); rene.schoenegg@h-och.ch (R.S.); 2 Institute of Pathology, Stadtspital Zürich, 8063 Zürich, Switzerland; 3 Division of Endocrinology and Diabetes, HOCH Health Ostschweiz, 9007 St. Gallen, Switzerland; frida.renstroem@h-och.ch (F.R.); barbara.bischofberger-baumann@h-och.ch (B.B.-B.); stefan.bilz@h-och.ch (S.B.); 4 Division of General Internal Medicine, HOCH Health Ostschweiz, 9007 St. Gallen, Switzerland; michael.braendle@h-och.ch

**Keywords:** thyroid nodule, thyroid cancer, fine-needle aspiration, molecular testing, seven-gene panel

## Abstract

Risk stratification of thyroid nodules is mainly based on ultrasound examination and fine-needle aspiration (FNA) cytology findings. Molecular testing has increasingly been added to the workup to improve risk of malignancy (ROM) estimation. Here, we evaluated the diagnostic performance of alterations in seven genes, including point mutations in *BRAF*, *HRAS*, *KRAS*, and *NRAS* as well as *RET/PTC1*, *RET/PTC3*, and *PAX8/PPARγ* fusions in 849 FNA samples with cytopathologically indeterminate Bethesda categories (III, IV, and V). In 20.14% of samples, at least one gene alteration was detected, with *NRAS* mutations and the *BRAF* V600E variant occurring most frequently. For 636 of the thyroid nodules, surgical follow-up was available, with a malignancy rate of 22.64%. *BRAF* V600E mutations and *RET/PTC1* fusions were associated with a ROM of 100%, *RAS* mutations with 13.64%, and *PAX8/PPARγ* fusions with 60.00%. Depending on the Bethesda category, the positive predictive value for malignancy of the seven-gene panel ranged between 18.18% (Bethesda III) and 91.07% (Bethesda V), while the negative predictive value ranged between 93.92% (Bethesda III) and 24.14% (Bethesda V). In conclusion, molecular testing with the seven-gene panel can improve ROM estimation in cytopathologically indeterminate thyroid nodules, but its clinical utility depends on the detected gene alteration.

## 1. Introduction

The incidence of thyroid cancer has been increasing worldwide over the past few decades [1]. Switzerland is no exception to this trend: According to the Swiss National Institute for Cancer Epidemiology and Registration (NICER), it rose from 5.13 per 100,000 person-years to 12.02 in women and from 2.76 to 5.13 in men during the last forty years [2]. As in other high-income countries, most of this increase is attributed to overdiagnosis [3,4,5,6]. Distinguishing malignant nodules from the more common benign ones can be challenging, especially considering that 2–6% of the population have palpable thyroid nodules, of which only up to 13% are malignant [7,8]. With high-frequency ultrasound (US) examination, nodules can be detected in as many as 68% of patients [9]. Established clinical methods to estimate the risk of malignancy (ROM) include patient history and clinical examination, measurement of thyroid-stimulating hormone levels, thyroid scintigraphy, and US evaluation of the neck region. Multiple US-based risk stratification systems for thyroid nodules are available that differ in methodological approach and diagnostic performance [10]. If suspicious US features are present and/or the nodule reaches a certain size, guidelines recommend US-guided fine-needle aspiration (FNA) to further assess the ROM [11,12].

Several systems for the cytopathological evaluation of thyroid aspirates have been developed over the years, with the Bethesda System for Reporting Thyroid Cytopathology (TBSRTC) being one of the more frequently used ones [13,14]. It is a useful tool to stratify nodules that are clearly benign or malignant with high certainty (malignancy risk of 2–7% in Bethesda category II [benign] and 97–100% in Bethesda category VI [malignant], respectively). However, up to 46% of all FNAs fall into the indeterminate Bethesda categories III (Atypia of Undetermined Significance), IV (Follicular Neoplasm), or V (Suspicious for Malignancy) with a ROM of 13–30%, 23–34%, and 67–83%, respectively. For a long time, the recommended management of nodules in these categories consisted of surveillance, repeat FNA, or diagnostic hemithyroidectomy, during which the thyroid lobe containing the nodule is surgically removed to acquire a definitive histopathological diagnosis [15,16]. This approach, however, often leads to over- or initial undertreatment of patients: If the nodule is diagnosed as benign after histopathological evaluation, surgery is not necessary for treatment; if it turns out to be malignant, a complete thyroidectomy might be indicated, depending on cancer type and staging. This two-step approach, therefore, increases patient burden and healthcare costs.

In recent guidelines, molecular testing has been added to the management recommendations for thyroid nodules with indeterminate cytology to improve treatment decisions [11,12]. Common genetic alterations associated with papillary thyroid carcinoma (PTC) include *BRAF*, *TP53*, and *TERT* promoter mutations as well as *BRAF* and *RET* fusions. Mutations in *RAS* genes and fusions involving *PPARγ* or *THADA* are found both in benign and malignant follicular neoplasms [17]. Commercially available molecular tests use different approaches to classify thyroid FNA samples and have shown solid performance in validation and real-world studies [18,19]. However, they were investigated and implemented mostly in North American cohorts, lacking validation data in other geographic regions and patient settings. Additionally, logistical and sustainability considerations in cases of centralized testing and unresolved issues of cost coverage prevent global use.

One of the first molecular tests investigated in thyroid nodules was a limited panel designed to detect hotspot point mutations in four genes (*BRAF*, *HRAS*, *KRAS*, and *NRAS*) and three gene fusions (*RET/PTC1*, *RET/PTC3*, and *PAX8/PPARγ*), often referred to as the seven-gene panel [20]. Due to its high initially reported specificity (96–99%) and positive predictive value (PPV) (87–95%), it has been proposed as a rule-in test to identify thyroid nodules with an increased ROM among those with an indeterminate Bethesda category, thereby triaging them towards surgical treatment [21]. Our objective was to assess the performance of this molecular test for preoperative risk stratification of thyroid nodules with indeterminate Bethesda categories (Bethesda III, IV, and V) in our institution in Eastern Switzerland. While the panel only reached a PPV of 47%, *BRAF* V600E mutations and *RET/PTC1* fusions were associated with a 100% ROM independent of Bethesda category. The seven-gene panel can therefore assist in identifying cancer among cytopathologically indeterminate thyroid nodules, but its clinical usefulness depends on the detected gene alteration.

## 2. Results

### 2.1. Cytological Findings

**Figure 1:**
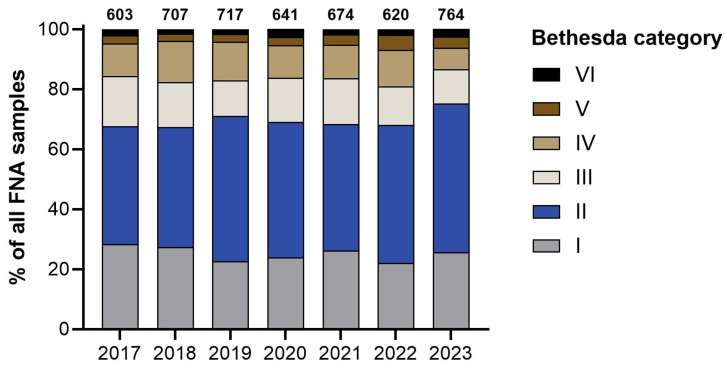
Yearly frequency distribution of Bethesda categories. Relative frequency of each Bethesda category in fine-needle aspiration (FNA) samples of thyroid nodules evaluated according to The Bethesda System for Reporting Thyroid Cytopathology (TBSRTC) during the seven-year study period. The total number of FNA samples in each year is shown above the bars.

First, we evaluated the distribution of Bethesda categories in 4726 FNA samples obtained from thyroid nodules over a seven-year period. The cumulative frequency rates for the Bethesda categories were as follows: Bethesda I, 25.06%; Bethesda II, 44.63%; Bethesda III, 13.90%; Bethesda IV, 11.16%; Bethesda V, 3.18%; Bethesda VI, 2.07%. The data was also analyzed per year, indicating the frequency distribution of the six Bethesda categories, including Bethesda III, IV, and V, to be relatively stable over time (Figure 1).

### 2.2. Results of Molecular Testing

Molecular testing with the seven-gene panel was routinely performed on FNA samples classified as Bethesda III, IV, or V during this study. The demographic characteristics of patients and their nodule characteristics are shown in Table A1 and Table A2, respectively. Of the 849 tests carried out, 781 (91.99%) had conclusive results. In the remaining 68 cases (8.01%), testing was inconclusive due to insufficient quantity or quality of extracted DNA and/or RNA, as indicated by the housekeeping gene control. In 20.14% of tests, a gene alteration could be detected (Table 1). *NRAS* point mutations were the most frequent (47.95%), followed by the *BRAF* V600E variant (31.58%) and *HRAS* point mutations (11.11%). Other gene alterations were less common, with individual frequencies below 5% of positive test results. No *RET/PTC3* fusions were detected. In one case, both an *HRAS* point mutation and a *PAX8/PPARγ* fusion were found in the same FNA sample.

**Figure 2:**
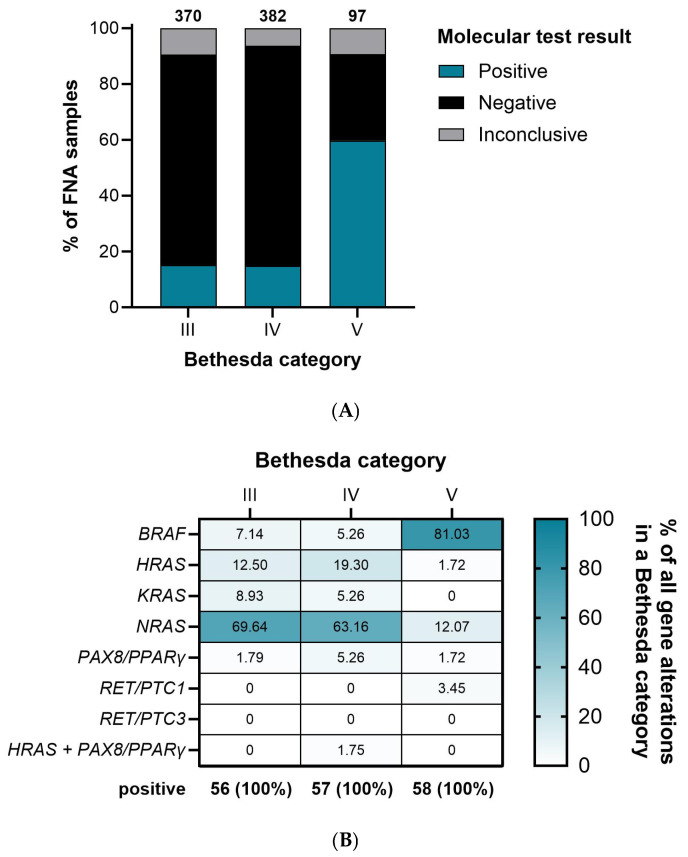
Molecular testing results with the seven-gene panel in Bethesda categories III, IV and V thyroid nodule FNA samples, stratified by Bethesda category. (**A**) Relative frequencies of molecular test results in Bethesda categories III, IV, and V thyroid nodules. The total number of FNA samples in each Bethesda category is shown above the bars. (**B**) Frequency of gene alterations by Bethesda category (III, IV, and V). Color intensity correlates with the frequency of positive test results in each category. Numbers below each column indicate the total number of positive test results.

A gene alteration was detected in approximately 15% of samples with Bethesda categories III or IV. In the Bethesda category V samples, the frequency of positive molecular test results was significantly higher, with 59.79% (Figure 2A). *BRAF* V600E variants and *RET/PTC1* fusions occurred mainly in FNA samples of Bethesda category V. In contrast, *RAS* mutations and *PAX8/PPARγ* fusions were mostly detected in Bethesda categories III and IV (Figure 2B). The rate of inconclusive testing was comparable between Bethesda categories and ranged between 6.28% and 9.46%.

### 2.3. Risk of Malignancy According to Bethesda Category

**Figure 3:**
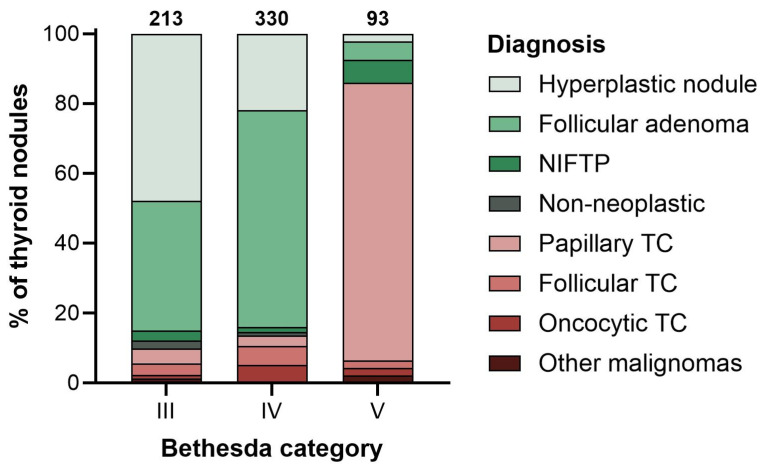
Histopathological findings in Bethesda categories III, IV, and V thyroid nodules with surgical follow-up, stratified by Bethesda category. Relative frequencies of histopathological diagnoses are shown for Bethesda categories III, IV, and V. ‘Other malignomas’ include anaplastic thyroid carcinoma, medullary thyroid carcinoma, and malignant teratoma. The total number of thyroid nodules in each Bethesda category is shown above the bars. NIFTP, non-invasive follicular neoplasm with papillary-like nuclear features; TC, thyroid carcinoma.

Surgical follow-up (hemithyroidectomy, total thyroidectomy, or thyroid isthmus resection) was available for 636 of 849 thyroid FNA samples with molecular testing (74.91%). A total of 144 thyroid nodules (22.64%) were classified as malignant after histopathological evaluation. In line with current practice [14], non-invasive follicular thyroid neoplasms with papillary-like nuclear features (NIFTP) were considered indolent and therefore benign for all analyses. Malignancy rates for the different Bethesda categories were as follows: Bethesda III, 8.92%; Bethesda IV, 13.64%; Bethesda V, 86.02% (Figure 3). Among malignant tumors, PTC was the most prevalent tumor type with 93 cases (64.58%), followed by follicular thyroid carcinoma (FTC) with 27 cases (18.75%) and oncocytic thyroid carcinoma with 20 cases (13.89%). Very rare tumor types detected in the cohort were medullary carcinoma (two cases, 1.42%), anaplastic carcinoma (one case, 0.71%), and malignant teratoma (one case, 0.71%) (Table A3).

### 2.4. Risk of Malignancy According to Individual Gene Alteration

**Figure 4:**
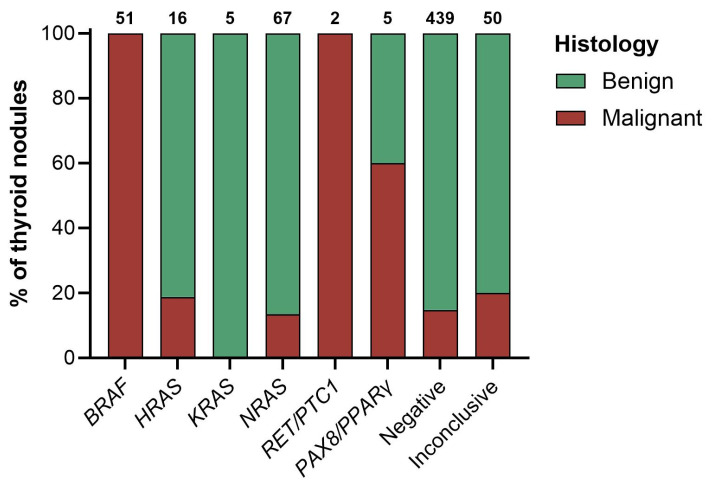
Relative risk of malignancy (ROM) after detection of individual gene alterations. The relative proportion of benign and malignant nodules after histopathological evaluation of Bethesda categories III, IV, and V thyroid nodules is shown, stratified by molecular testing result. The total number of thyroid nodules with each gene alteration is shown above the bars. One nodule with both an *HRAS* point mutation and a *PAX8/PPARγ* fusion detected is not included in this graph.

For 147 (23.11%) of the nodules with surgical follow-up, preceding molecular testing of the corresponding FNA sample had been positive. The ROM varied greatly depending on the detected gene alteration: *BRAF* V600E, 100%; *HRAS* mutations, 18.75%; *KRAS* mutations, 0.00%; *NRAS* mutations, 13.43%; *RET/PTC1* fusions, 100%; and *PAX8/PPARγ* fusions, 60.00% (Figure 4). After a negative or inconclusive molecular test result, 14.81% and 20.00% of nodules were malignant, respectively.

All nodules with the *BRAF* V600E variant were diagnosed as PTC. *HRAS* mutations were found in benign nodules or FTC, as well as in the single medullary carcinoma of the cohort. *KRAS* mutations were only present in benign nodules, as well as in a case without a clearly distinguishable nodule, where only fibrotic thyroid tissue could be seen. *NRAS* mutations were mostly found in follicular adenomas and hyperplastic nodules, as well as in some NIFTPs, PTCs, and FTCs. The two nodules with a *RET/PTC1* fusion were both classic PTCs. Similar to *NRAS* mutations, *PAX8/PPARγ* fusions were found in benign nodules, PTCs, and FTCs. The nodule with both an *HRAS* point mutation and a *PAX8/PPARγ* fusion detected was diagnosed as a minimally invasive FTC (Table A4).

### 2.5. Hyperplastic Nodules with Gene Alterations

Interestingly, in 12 cases with a detected *RAS* mutation in the FNA sample, the histopathological diagnosis after resection was a benign hyperplastic nodule (Table A4). To verify this finding, the cases were re-evaluated by a second board-certified pathologist blinded to the initial diagnosis. The diagnosis was confirmed for all 12 nodules. Additionally, molecular testing was repeated on the formalin-fixed paraffin-embedded (FFPE) resection specimens, and the specific gene variants were identified by pyrosequencing (Table S1). In nine of the 12 cases, the mutations initially detected in the FNA samples were confirmed. Pyrosequencing was inconclusive in two cases and negative in one case.

### 2.6. Bethesda VI Nodules

To further evaluate the specificity of the seven-gene panel and establish the correlation between clearly malignant FNA samples and the occurrence of gene alterations, we also performed molecular testing on 63 FNA samples categorized as Bethesda VI. A point mutation or fusion was detected in 54 cases (85.71%), five molecular tests (7.94%) were negative, and four molecular tests (6.35%) did not show a conclusive result. Almost all detected variants were *BRAF* V600E (51 cases, 94.44%), with the other three cases showing *RET/PTC* fusions (two *RET/PTC1* fusions, one *RET/PTC3* fusion). In addition, 61 of the cases had surgical follow-up, which resulted in a malignancy rate of 100% with the histological diagnoses of 59 PTCs (96.72%), one medullary carcinoma (1.64%), and one anaplastic carcinoma (1.64%). In this Bethesda category, all cases with a positive molecular test result proved to be malignant after resection (Table A5).

### 2.7. Diagnostic Performance of the Seven-Gene Panel in FNA Samples with Indeterminate Cytology

**Figure 5:**
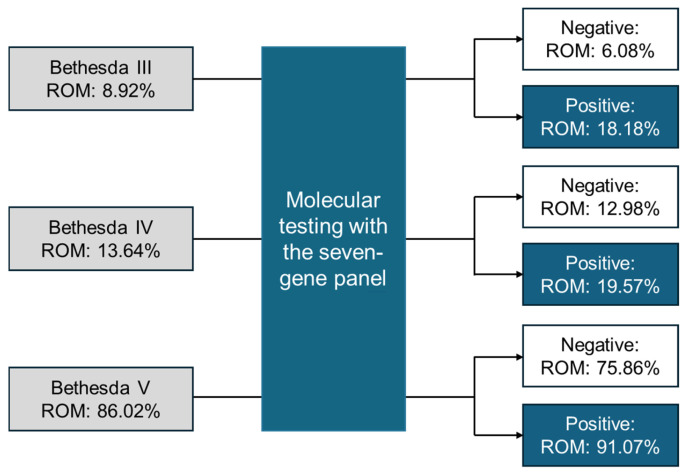
Pre- and post-test ROM of Bethesda categories III, IV, and V thyroid nodule FNA samples after molecular testing with the seven-gene panel. ROM values in Bethesda categories III, IV, and V thyroid nodule FNA samples are shown before and after molecular testing with the seven-gene panel, depending on the test result.

The primary aim of this study was to evaluate the capacity of the seven-gene panel to accurately separate benign nodules and NIFTP from cancer in FNA samples with indeterminate cytology (Bethesda categories III, IV, and V). Surgical follow-up was available for 586 FNA samples with these Bethesda categories and conclusive molecular testing. Table 2 summarizes the sensitivity, specificity, negative predictive value (NPV), and PPV of the seven-gene panel for the identification of malignant lesions. For all three Bethesda categories combined, the NPV of the panel was 85.19% (95% confidence interval [CI] [81.52%, 88.38%]) and the PPV was 46.58% (95% CI [38.29%, 55.01%]). In the individual Bethesda categories, the NPV ranged between 93.92% (95% CI [88.77%, 97.18%], Bethesda III) and 24.14% (95% CI [10.30%, 43.54%], Bethesda V), while the PPV ranged between 18.18% (95% CI [8.19%, 32.71%], Bethesda III) and 91.07% (95% CI [80.38%, 97.04%], Bethesda V). An overview of pre- and post-test ROM after molecular testing, depending on the Bethesda category, is shown in Figure 5.

### 2.8. Predictive Power of Patient and Nodule Characteristics in Combination with RAS-like Mutations

In our cohort, the *BRAF* V600E variant and *RET/PTC1* fusion were associated with a 100% ROM, clearly indicating that nodules with these gene alterations should be managed actively regardless of other nodule characteristics (Table A6). To facilitate clinical decision-making for nodules lacking such unequivocal indicators of malignancy, we evaluated whether adding information about the presence or absence of the other gene alterations in the seven-gene panel increased the predictive power of a model combining several patient and nodule characteristics.

**Figure 6:**
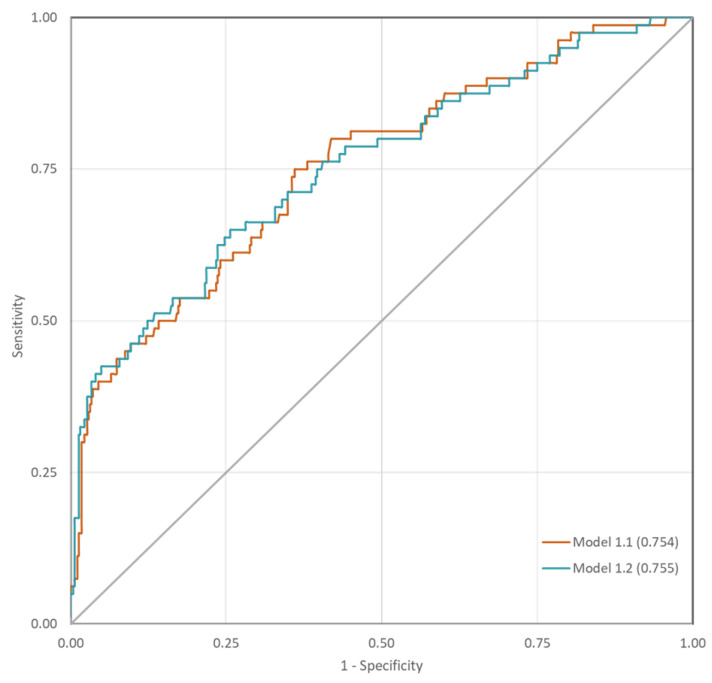
Receiver operating characteristic/area under the curve (ROCAUC) plot comparing multivariable regression models for Bethesda categories III, IV, and V thyroid nodules based on the core characteristics with or without the inclusion of genetic information. Model 1.1 comprises the variables age, sex, size on US, and Bethesda category (reference category: III). Model 1.2 is the same as Model 1.1 with added information about the presence of *HRAS* point mutations, *NRAS* point mutations, and *PAX8/PPARγ* fusions. The ROCAUC of the two models did not differ significantly from each other (Model 1.1, 0.754, 95% CI [0.693, 0.816] vs. Model 1.2, 0.755, 95% CI [0.692, 0.818], *p* = 0.98).

We based the model on a cohort of 533 Bethesda categories III, IV, and V thyroid nodules with surgical follow-up after excluding nodules with a *BRAF* V600E variant, a *RET/PTC1* fusion, or an inconclusive molecular test result. Thyroid nodules with *KRAS* point mutations were also excluded, as these mutations occurred only in benign nodules. In the final cohort, 81 nodules (15.20%) were malignant. Univariate analysis revealed that among the core characteristics (age, sex, size on US, Bethesda category, molecular test result), only age, Bethesda category, and the presence of a *PAX8/PPARγ* fusion were significantly associated with malignancy (Table 3). Adding the genetic information to a regression model comprising the other core characteristics (age, sex, size on US, Bethesda category [reference category: III]) did not improve the predictive power of a multivariable regression model (Receiver Operating Characteristic/Area Under the Curve [ROCAUC] 0.754, 95% CI [0.693, 0.816] vs. 0.755, 95% CI [0.692, 0.818], *p* = 0.98) (Figure 6).

**Figure 7:**
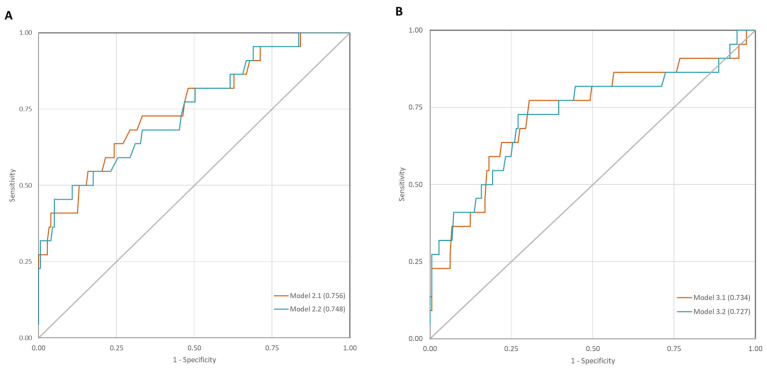
ROCAUC plot comparing multivariable regression models for Bethesda categories III, IV, and V thyroid nodules based on the extended characteristics with or without the inclusion of genetic information. (**A**) Model 2.1 comprises the variables age, sex, margin (lobular vs. not), texture (solid vs. not), echogenicity (marked hypoechoic vs. not), focal changes (microcalcifications, macrocalcifications, and not classifiable), and Bethesda (5 vs. not). Model 2.2 is the same as Model 2.1 with added information about the presence of *HRAS* point mutations, *NRAS* point mutations, and *PAX8/PPARγ* fusions. The ROCAUC of the two models did not differ significantly from each other (Model 2.1, 0.756, 95% CI [0.639, 0.734] vs. Model 2.2, 0.748, 95% CI [0.630, 0.867], *p* = 0.67). (**B**) Model 3.1 comprises the variables age, sex, EU-TIRADS (4 vs. 3, 5 vs. 3), and Bethesda (5 vs. not). Model 3.2 is the same as Model 3.1 with added information about the presence of *HRAS* point mutations, *NRAS* point mutations, and *PAX8/PPARγ* fusions. The ROCAUC of the two models did not differ significantly from each other (Model 3.1, 0.734, 95% CI [0.605, 0.863] vs. Model 3.2, 0.727, 95% CI [0.592, 0.863], *p* = 0.78).

We repeated the analysis described above with a smaller set of nodules, for which additional clinical and US information was available. Of the 199 nodules included, 22 (11.06%) were malignant. In a univariate analysis of extended characteristics, a lobulated margin, marked hypoechogenicity, Bethesda category V, and the detection of a *PAX8/PPARγ* fusion were significantly associated with malignancy (Table 4). Several other characteristics known to be correlated with an increased ROM of thyroid nodules, such as microcalcifications detected in US and a high European Thyroid Imaging Reporting and Database System (EU-TIRADS) class, showed a trend towards correlation. Adding the results of molecular testing to a regression model including the identified characteristics did not improve the predictive power of the multivariable regression model, neither using individual US characteristics (ROCAUC 0.756, 95% CI [0.639, 0.734] vs. 0.748, 95% CI [0.630, 0.867], *p* = 0.67; Figure 7A) nor using the EU-TIRADS class as a summarized variable (ROCAUC 0.734, 95% CI [0.605, 0.863] vs. 0.727, 95% CI [0.592, 0.863], *p* = 0.78; Figure 7B). Comparable results were obtained for the subgroup of Bethesda III/IV nodules (Tables S2 and S3, Figures S1 and S2).

## 3. Discussion

In this study, we evaluated the capacity of a seven-gene panel testing approach to improve the estimation of ROM in indeterminate thyroid FNA samples in our setting in Eastern Switzerland. With a cohort of 849 FNA samples and a 75% rate of surgical follow-up, it constitutes the largest series of thyroid nodule FNA samples correlated with seven-gene molecular testing and histological diagnosis to date. In prior investigations, authors reported varying performance parameters, with PPVs ranging between 19% and 100% and NPVs ranging between 62% and 91% for indeterminate FNA samples [20,21,22,23,24,25,26,27]. The NPV in our cohort (85.18%, 95% CI 81.52–88.38%) was comparable to those previously found, while the PPV was rather low at 46.58% (95% CI 38.29–55.01%). PPV and NPV are parameters influenced by the pre-test probability of the tested condition: A lower pre-test probability tends to lead to a lower PPV and higher NPV if other test parameters remain the same [28]. Consequently, the malignancy rates in Bethesda III and IV samples of our cohort, which were at the lower end of the expected range according to TBSRTC 2017, with 8.92% and 13.64%, respectively, might have led to a lower PPV of a positive molecular test result in these Bethesda categories. Another possible reason for the lower PPV is the reclassification of the encapsulated follicular variant of PTC to NIFTP in 2016, implemented after most of the studies investigating the seven-gene panel were published [29]. This change, aiming to better reflect the indolent behavior of this entity, lowers the ROM for all Bethesda categories and therefore the pre-test probability of malignancy before molecular testing [13,14,30,31,32]. Additionally, NIFTPs often harbor *RAS* mutations [33]; if they are considered benign, the PPV for malignancy of any molecular test, including *RAS* mutations, will decrease. Similarly, the benign nodules in our cohort contained a relatively large fraction of follicular adenomas compared with most other studies, which also lowers the PPV due to the high frequency of *RAS* mutations in these tumors [34].

We observed the same distinct difference in PPV depending on the detected gene alteration and mutation group as initially described by The Cancer Genome Atlas Project [35]. All resected thyroid nodules with *BRAF*-like genomic changes (*BRAF* V600E mutations and *RET/PTC1* fusions) were diagnosed as PTC, resulting in a PPV of 100% irrespective of the Bethesda category of the FNA sample. For *RAS*-like gene alterations (*HRAS*, *KRAS*, *NRAS* mutations and *PAX8/PPARγ* fusions), PPVs ranged between 0% for *KRAS* mutations and 60% for *PAX8/PPARγ* fusions. In a systematic review including studies published until 2018, Goldner et al. found similar PPVs for the *BRAF* V600E variant, *RET/PTC1* fusions, and *PAX8/PPARγ* fusions detected with the seven-gene panel, while the PPV of *RAS* mutations was higher (66%) compared to our results [36]. In more recent studies, authors reported PPVs for *RAS* mutations ranging between 29% and 50% [26,27,37]. The varying performance of *RAS* mutations as an indicator of malignancy is likely due to their occurrence in both malignant and benign neoplasms, resulting in a considerable impact of the cohort’s tumor type composition on the predictive power of these gene alterations [34]. In line with previous reports, we detected *RAS* point mutations not only in FTCs, but also in a significant number of follicular adenomas and hyperplastic nodules.

Other, more extensive panels—such as the Afirma Genomic Sequencing Classifier (GCS), ThyroSeq v.3, and the ThyGeNEXT/ThyraMIR assay—have demonstrated better performance parameters with NPVs ranging between 95% and 97% and PPVs between 47% and 74% [19]. However, the seven-gene panel has several advantages: It does not require a dedicated pass during FNA and functions with very low input concentrations of DNA and RNA, as demonstrated by our dilution series during the technical validation (Tables S4 and S5). It can be implemented in-house with appliances already present in most molecular pathology labs instead of being sent abroad for centralized testing, reducing processing time and logistical effort, as well as being more sustainable. The seven-gene panel is also considerably less expensive. Its clinical usefulness, however, depends on whether the results are interpreted accurately in the local context. Thyroid cancer prevalence in the population, referral practices, and institutional classification habits influence the probability for malignancy in individual Bethesda categories and, consequently, the performance of ancillary molecular testing.

In our study, a *BRAF* V600E mutation or *RET/PTC1* fusion was diagnostic for PTC, independent of clinical characteristics or cytopathological classification. Consequently, these gene alterations might be suited to detect malignant nodules among Bethesda III and IV nodules in our setting and to guide clinical management towards surgery (i.e., rule-in strategy), with its extent depending on other risk factors such as nodule size and lymph node involvement. Furthermore, additional preoperative testing for *TERT* promoter mutations in thyroid nodules with a *BRAF* V600E mutation might also impact management due to the worse prognosis in dual-positive cases [38]. In contrast, the detection of a *RAS* mutation as an isolated finding did not result in a PPV that would justify oncologic resection of the thyroid gland. Thyroid nodules with these mutations were mostly benign, with only 13.6% diagnosed as thyroid carcinomas. Increasing evidence points toward *RAS* mutations being a feature of benign or low-risk follicular-patterned neoplasms [34,39]. However, they are also correlated with larger size and faster growth in thyroid nodules with benign cytology, possibly requiring surgical removal nonetheless [40]. A limited surgical approach might therefore be the most suitable choice if such a mutation is found [41]. Bethesda V nodules with a negative molecular test result showed an NPV of 24.14%, which is not sufficiently high to waive the recommended surgical management in this Bethesda category [11,12]. Although not the focus of our study, we did observe that patients were more likely to be treated surgically after a positive molecular test result, and the rate of malignancy after both hemithyroidectomy and total thyroidectomy was higher compared with those with negative molecular testing (Table A7). The frequency of completion thyroidectomies after a hemithyroidectomy of a malignant nodule was similar in both groups.

Our study has several limitations: Despite the very high rate of surgical follow-up, not all thyroid nodules that underwent molecular testing received a definite histopathological diagnosis. Thyroid nodules with a missing gold standard were more likely to have low-risk features (Table S6), thus possibly artificially increasing the PPV and decreasing the NPV of the seven-gene panel. Our cohort included only a few indeterminate nodules with *RET/PTC1* fusions and lacked nodules with *RET/PTC3* fusions, limiting our ability to judge the predictive power for malignancy of these gene alterations. Additionally, as the frequency distribution and ROM of Bethesda categories often vary between geographical regions and different pathology institutes, our findings might not be directly applicable to other institutions [42,43,44].

In conclusion, the seven-gene panel investigated here can improve clinical management of indeterminate thyroid nodules by identifying thyroid carcinomas among cytopathologically inconspicuous FNA samples via the detection of *BRAF*-like gene alterations. These nodules would otherwise initially be treated with surveillance and repeat FNAs. Patients could therefore benefit from earlier diagnosis and a definite surgical approach from the outset. *RAS*-like mutations do not show the same predictive power, even in combination with other patient and nodule characteristics, limiting their impact on thyroid nodule management. It is paramount that treating physicians are aware of the institute-specific characteristics of any molecular test that might influence patient treatment.

## 4. Materials and Methods

### 4.1. Study Design and Patient Cohort

This single-center observational cohort study assessed a consecutive series of thyroid nodule FNA samples processed and evaluated at the Institute of Pathology, Cantonal Hospital St. Gallen, between 1 December 2016 and 31 December 2023. Patients were included if their FNA samples were categorized as Bethesda III, IV, V, or VI by a board-certified cytopathologist and if molecular testing with the seven-gene panel was carried out subsequently.

### 4.2. Clinical Characteristics and US Characteristics

The following clinical information was collected from all patients: age, sex, nodule number, nodule location (right thyroid lobe, left thyroid lobe, or isthmus), and nodule diameter on US (if available). For a subgroup of patients, additional clinical characteristics (risk factors, known thyroid conditions, and symptoms) and individual US criteria were available. For these patients, the neck region was assessed using a high-resolution US color Doppler instrument by or under the supervision of a board-certified endocrinologist. Prespecified US characteristics of thyroid nodules were collected, including width, height, and length of the nodule; margin (smooth, irregular, lobulated, or spiculated); composition (solid, partially cystic, mostly cystic with solid parts, purely cystic, spongiform, or nodular hyperplasia); echogenicity (hyper-, iso-, hypo-, or markedly hypoechoic); and focal changes (none, macrocalcifications, calcifications with a comet tail sign, microcalcifications, or not-classifiable calcifications). The nodules were then classified into four categories according to the guidelines for US malignancy risk stratification of thyroid nodules in adults proposed by the European Thyroid Association: EU-TIRADS 2 (benign category), EU-TIRADS 3 (low-risk category), EU-TIRADS 4 (intermediate-risk category), and EU-TIRADS 5 (high-risk category) [45].

### 4.3. Cytological Evaluation

US-guided FNA was performed after clinical establishment of the indication by the treating physician. Conventional smears were prepared right after the aspiration and fixed in Delaunay solution. The remaining material was collected and conserved in ThinPrep CytoLyt solution (Hologic Inc., Marlborough, MA, USA) as liquid-based cytology material. A single ThinPrep slide was prepared using a ThinPrep 2000 instrument (Hologic Inc.) according to the manufacturer’s instructions. The remaining liquid-based material was stored at 4 °C for molecular testing after cytopathological evaluation of the sample, if indicated. Both ThinPrep slides and conventional smears were stained with the Papanicolaou method. Stained slides were then reviewed by a board-certified cytopathologist. Results were reported using the 2017 Bethesda System for Reporting Thyroid Cytopathology [46], which applies six diagnostic categories: (i) nondiagnostic or unsatisfactory (Bethesda I); (ii) benign (Bethesda II); (iii) atypia of undetermined significance or follicular lesion of undetermined significance (Bethesda III); (iv) follicular neoplasm or suspicious for a follicular neoplasm (Bethesda IV); (v) suspicious for malignancy (Bethesda V); and (vi) malignant (Bethesda VI). In difficult cases, a second board-certified cytopathologist was involved to reach a consensus diagnosis.

### 4.4. Molecular Testing with the Seven-Gene Panel

Molecular testing was reflexively performed following the diagnosis of Bethesda categories III, IV, V, or VI as part of routine clinical practice if residual liquid-based FNA material was available. Total nucleic acids were extracted from liquid-based FNA samples using a Maxwell RSC instrument (Promega, Madison, WI, USA) with the Maxwell RSC Viral Total Nucleic Acid Purification Kit (Promega) according to the manufacturer’s instructions. Molecular testing of DNA was performed using the Thyroid Cancer Mutation Analysis Kit (EntroGen, Woodland Hills, CA, USA), which includes point mutations in *BRAF* (V600E), *KRAS* codons 12, 13, and 61 (G12D, G12A, G12V, G12S, G12R, G12C, G13D, Q61E, Q61K, Q61L, Q61R, Q61P, Q61H, Q61H), *NRAS* codons 12, 13, 61, and 146 (G12D, G12S, G12C, G13R, G13V, Q61K, Q61L, Q61R, Q61H, A146T), and *HRAS* codons 12, 13, and 61 (G12V, G13R, Q61R). The Thyroid Cancer Fusion Gene Detection Kit (EntroGen) was used to detect *RET/PTC1* inv(10) (q11q21), *RET/PTC3* inv(10)(q11q11), and *PAX8/PPARγ* t(2;3)(q13;p25) fusions in extracted RNA, with reverse transcription and PCR amplification performed in a single step. For quantitative polymerase chain reaction (qPCR), a C1000 Touch Thermal Cycler and a CFX96 Touch Real-Time PCR Detection System (both Bio-Rad Laboratories, Hercules, CA, USA) were used. A sample was classified as positive for the seven-gene panel if analysis of both DNA and RNA yielded conclusive results and at least one gene alteration was detected. A test was considered inconclusive if analysis of DNA, RNA, or both failed due to insufficient quantity or quality as indicated by the housekeeping gene control.

### 4.5. Technical Validation of the Seven-Gene Panel

To establish the minimal input amount of DNA and RNA required for the seven-gene panel, two dilution series were carried out. For the DNA-based part of the panel (*BRAF*, *HRAS*, *KRAS*, and *NRAS* point mutations), the AcroMetrix Oncology Hotspot Control (Thermo Fisher Scientific, Waltham, MA, USA) was used undiluted, at a 1:10 dilution, and at a 1:100 dilution. DNA concentrations were measured on a Qubit 2.0 Fluorometer (Thermo Fisher Scientific) at 1.96 ng/µL, 0.290 ng/µL, and not measurable, respectively. The qPCR curves showed the expected shift to the right with every dilution, and molecular testing was considered positive in all three cases (Table S2). For the RNA-based part of the panel (*RET/PTC1*, *RET/PTC3*, and *PAX8/PPARγ* fusions), the positive control included in the Thyroid Cancer Fusion Gene Detection Kit (EntroGen) was tested undiluted, at 1:10, and at 1:100 dilution. Measurement of RNA concentrations was not possible as the positive control for the kit is synthetically produced with a prespecified CT range. In this case as well, the qPCR curves shifted to the right with every dilution, and the molecular test was positive in all three cases (Table S3). Molecular testing of FNA samples was therefore performed without applying minimal DNA/RNA input requirements.

### 4.6. Histological Evaluation

Thyroid lobectomy, thyroid isthmus resection, and total thyroidectomy specimens were fixed in buffered formalin. Macroscopic evaluation and processing were performed according to in-house standards. Tumors with a diameter of up to 4 cm were embedded completely. For larger tumors, at least two tissue blocks were prepared per centimeter of tumor diameter. In such cases, tumor tissue was preferentially sampled and embedded from the periphery of thyroid nodules to identify capsular penetration or vascular invasion. Sections stained with hematoxylin and eosin were evaluated by board-certified pathologists, and additional conventional and immunohistochemical stains were performed if deemed necessary. Diagnostic classification of tumors was done according to the World Health Organization Classification of Tumors of Endocrine Organs (4th Edition). In difficult cases, a second board-certified pathologist was involved to reach a consensus diagnosis. Pathologists were not blinded to the molecular testing result.

### 4.7. Confirmation of Detected Gene Alterations on Resection Material

To confirm mutations detected in FNA samples of thyroid nodules that were diagnosed as hyperplastic nodules after surgery, hotspot mutations were additionally identified by pyrosequencing. DNA was extracted from FFPE tissue blocks using the Maxwell RSC DNA FFPE kit (Promega) according to the manufacturer’s instructions, and protocols were adapted in-house to optimize DNA yield and purity. Target regions were amplified by PCR, followed by pyrosequencing on the PyroMark Q24 platform (QIAGEN, Hilden, Germany), in accordance with the manufacturer’s instructions and in-house adapted protocols. The analysis covered hotspot mutations in the following regions: *NRAS* exon 2 (codons 12 and 13), *NRAS* exon 3 (codons 58–61), *KRAS* exon 2 (codons 12 and 13), and *HRAS* exon 3 (codon 61).

### 4.8. Statistical Analysis and Figures

The performance of the seven-gene panel was evaluated by calculating sensitivity, specificity, NPV, and PPV using the histopathological diagnosis of the thyroid nodules as the gold standard. Statistical analysis was performed with R version 4.5.2 (R Foundation for Statistical Computing, Vienna, Austria) [47] and R Studio version 2025.09.2+418 (Posit PBC, Boston, MA, USA). Probability (*p*) values < 0.05 were considered statistically significant. Figures were made with GraphPad Prism software, version 10.6.1 (GraphPad Software, Boston, MA, USA).

Univariate logistic regression was used to determine if individual nodule characteristics were associated with malignancy. Characteristics with a *p*-value < 0.10 were combined in a multivariable logistic regression model, adjusted for age and sex. An extended model, additionally including *NRAS* mutations, *HRAS* mutations, and *PAX8/PPARγ* fusions, was applied to assess if genetic information collected using the seven-gene panel could improve the prediction of malignancy. Due to perfect predictor separation, *BRAF* V600E, *KRAS* mutations, and *RET/PTC1* fusions were excluded from the analysis. Statistically significant differences between ROCAUCs were determined using DeLong’s test [48]. This analysis was also performed with an extended set of patient and nodule characteristics in a smaller subcohort, once with individual US characteristics and once with the EU-TIRADS class as a summarized variable.

## Figures and Tables

**Table 1:** Molecular testing results with the seven-gene panel in Bethesda categories III, IV, and V thyroid nodule FNA samples.

Test Result	Number (%), Total n = 849
Positive	171 (20.14%)
Negative	610 (71.85%)
Inconclusive	68 (8.01%)
**Gene alteration detected**	**Number (% of detected), total n = 171**
*BRAF* V600E	54 (31.58%)
*HRAS* point mutation	19 (11.11%)
*KRAS* point mutation	8 (4.68%)
*NRAS* point mutation	82 (47.95%)
*RET/PTC1* fusion	2 (1.17%)
*RET/PTC3* fusion	0 (0%)
*PAX8/PPARγ* fusion	5 (2.92%)
*HRAS* point mutation + *PAX8/PPARγ* fusion	1 (0.58%)

**Table 2:** Diagnostic performance of the seven-gene panel in predicting malignancy in Bethesda categories III, IV, and V thyroid nodules.

Bethesda (n)	Sensitivity (95% CI)	Specificity (95% CI)	NPV (95% CI)	PPV (95% CI)
III + IV + V (585)	51.13% (42.31–59.89%)	82.74% (78.94–86.11)	85.19% (81.52–88.38%)	46.58% (38.29–55.01%)
III + IV (500)	28.33% (17.45–41.44%)	83.41% (79.60–86.76%)	89.51% (86.13–92.31%)	18.89% (11.41–28.51%)
III (192)	47.06% (22.98–72.19%)	79.43% (72.68–85.16%)	93.92% (88.77–97.18%)	18.18% (8.19–32.71%)
IV (308)	20.93% (10.04–36.04%)	86.04% (81.27–89.98%)	87.02% (82.34–90.84%)	19.57% (9.36–33.91%)
V (85)	69.86% (58.00–80.06%)	58.33% (27.67–84.83%)	24.14% (10.30–43.54%)	91.07% (80.38–97.04%)

NIFTPs were considered benign during the analysis. A positive molecular test result was considered a true positive in a histologically malignant nodule and a false positive in a benign nodule. A negative molecular test result was considered a true negative in a histologically benign nodule and a false negative in a malignant nodule. One malignant nodule with the detection of both an *HRAS* point mutation and a *PAX8/PPARγ* fusion was not included in this analysis. CI, confidence interval; NPV, negative predictive value; PPV, positive predictive value.

**Table 3:** Correlation of core characteristics with ROM in Bethesda categories III, IV, and V thyroid nodules with molecular testing of FNA samples and surgical follow-up (n = 533). Nodules with a *BRAF* V600E variant, a *RET/PTC1* fusion, *KRAS* point mutations, or an inconclusive molecular test result were excluded. Odds ratios were calculated with univariate logistic regression.

Characteristic	Odds	95% CI	*p*-Value
Age, years	0.98	0.97–1.00	0.032
Female sex (yes/no)	1.06	0.62–1.81	0.84
Size on US ^1^, mm	1.01	1.00–1.03	0.07
Bethesda IV vs. III	1.93	1.02–3.65	0.042
Bethesda V vs. III	27.08	11.30–64.92	<0.0001
*HRAS* point mutation	1.75	0.56–5.52	0.34
*NRAS* point mutation	0.85	0.40–1.79	0.67
*PAX8/PPARγ* fusion	11.68	2.10–64.87	0.005

^1^ Information was missing from nine patients.

**Table 4:** Correlation of extended characteristics with ROM in Bethesda categories III, IV, and V thyroid nodules with molecular testing of FNA samples and surgical follow-up (n = 199). Nodules with a *BRAF* V600E variant, a *RET/PTC1* fusion, *KRAS* point mutations, or an inconclusive molecular test result were excluded. Odds ratios were calculated with univariate logistic regression.

Characteristic	Odds	95% CI	*p*-Value
Age, years	0.98	0.95–1.01	0.10
Female sex (yes/no)	1.72	0.56–5.35	0.35
Height, mm	1.03	0.98–1.08	0.25
Length, mm	1.00	0.98–1.03	0.92
Width, mm	1.01	0.97–1.04	0.73
Taller than wide (yes/no)	1.01	0.22–4.70	0.99
Size on US, mm	1.00	0.98–1.03	0.85
Risk factors ^1^ (yes/no)	0.32	0.04–2.49	0.28
Known thyroid disease ^2^ (yes/no)	1.30	0.89–1.92	0.18
Symptoms ^3^ (yes/no)	1.37	0.54–3.47	0.50
Incidentaloma (yes/no)	0.60	0.22–1.61	0.31
**Margin**			
Sharp (vs. not)	0.36	0.11–1.21	0.10
Lobulated (vs. not)	4.15	1.16–14.84	0.029
**Texture**			
Solid (vs. not)	5.74	0.75–44.05	0.093
Partially cystic (vs. not)	0.20	0.03–1.54	0.12
**Echogenicity (categorical)**			0.026
Hyperechoic (vs. not)	1.36	0.16–11.83	0.78
Isoechoic (vs. not)	0.68	0.26–1.75	0.43
Hypoechoic (vs. not)	0.72	0.30–1.75	0.47
Marked hypoechoic (vs. not)	9.61	2.21–41.74	0.0025
**Focal changes (categorical)**			0.09
Microcalcifications (vs. no changes)	6.35	0.99–40.71	0.051
Macrocalcifications (vs. no changes)	9.53	0.57–159.31	0.12
Comet tail sign (vs. no changes)	0.95	0.12–7.90	0.96
Not classifiable (vs. no changes)	9.53	0.57–159.31	0.12
**EU-TIRADS, class**			0.053
4 (vs. 3)	0.67	0.22–2.04	0.48
5 (vs. 3)	2.63	0.90–7.71	0.08
**Bethesda, category**			0.0008
IV (vs. III)	1.56	0.53–4.63	0.42
V (vs. III)	34.50	5.29–224.77	0.0002
**Mutation (yes/no)**	1.50	0.51–4.39	0.46
*HRAS*	2.06	0.22–19.30	0.53
*NRAS*	0.64	0.14–2.90	0.56
*PAX8/PPARγ*	17.60	1.53–202.86	0.022

^1^ Risk factors include: family history of thyroid cancer in a first-degree relative, personal history of thyroid cancer, prior radiation therapy to the neck region, other radiation exposure, FDP-PET positivity of the nodule, and hereditary cancer syndromes such as MEN2. ^2^ Known thyroid diseases include: chronic autoimmune thyroiditis, Graves’ disease, euthyroid struma, struma with hyperthyroidism, and thyroid cancer. ^3^ Symptoms include: pain, difficulty swallowing, globus sensation, and hoarseness. EU-TIRADS, European Thyroid Imaging Reporting and Database System.

## Data Availability

All data generated or analyzed during this study are included in the published article and its Supplementary Information Files or are available from the authors upon request.

## Supplementary Materials

The following supporting information can be downloaded at: https://www.mdpi.com/article/10.3390/ijms27114990/s1.

## Abbreviations

The following abbreviations are used in this manuscript:

FNA	Fine-needle aspiration
ROM	Risk of malignancy
NICER	Swiss National Institute for Cancer Epidemiology and Registration
US	Ultrasound
TBSRTC	The Bethesda System for Reporting Thyroid Cytopathology
PTC	Papillary thyroid carcinoma
PPV	Positive predictive value
NIFTP	Non-invasive follicular thyroid neoplasm with papillary-like nuclear features
FTC	Follicular thyroid carcinoma
TC	Thyroid carcinoma
FFPE	Formalin-fixed paraffin-embedded
NPV	Negative predictive value
CI	Confidence interval
ROCAUC	Receiver operating characteristic/area under the curve
EU-TIRADS	European Thyroid Imaging Reporting and Database System
qPCR	Quantitative polymerase chain reaction
Cq	Quantification cycle
EKOS	Ethics Committee of Eastern Switzerland

## Appendix A

**Table A1:** Clinical characteristics of patients with Bethesda categories III, IV, and V thyroid nodules and molecular testing of FNA samples (n = 804).

Clinical Characteristic	Number (%)
**Sex**	
Female	580 (72.14%)
Male	224 (27.86%)
**Age** (in years; median [interquartile range])	56 (45–67)
Female	55 (44–67)
Male	59 (49–68)
**Referrer**	
In-house	549 (68.28%)
External	255 (31.72%)
**FNA performed by**	
Endocrinologist	358 (44.53%)
Cytopathologist	270 (33.58%)
Other	176 (21.89%)
**Number of nodules**	
One	760 (94.53%)
Two	43 (5.35%)
Three	1 (0.12%)

**Table A2:** Characteristics of Bethesda categories III, IV, and V thyroid nodules with molecular testing of FNA samples (n = 849).

Nodule Characteristic	Number (%)
**Localization**	
Right thyroid lobe	441 (52.07%)
Left thyroid lobe	365 (43.09%)
Isthmus	41 (4.84%)
**Size on US** (in mm; median [interquartile range])	23 (16–32)
**Bethesda category**	
III	370 (43.58%)
IV	382 (44.99%)
V	97 (11.43%)

US, ultrasound.

**Table A3:** Histopathological findings in Bethesda categories III, IV, and V thyroid nodules with molecular testing of FNA samples and surgical follow-up, stratified by Bethesda category (n = 636).

Histopathological Diagnosis	Bethesda III (n = 213)	Bethesda IV (n = 330)	Bethesda V (n = 93)
Hyperplastic nodule	103	72	2
Follicular thyroid adenoma	79	205	5
NIFTP	6	5	6
Papillary thyroid carcinoma (PTC)	9	10	74
Infiltrative follicular variant of PTC	4	7	21
Oncocytic PTC	0	1	1
Classic PTC	5	1	46
Encapsulated classic PTC	0	1	5
Tall cell PTC	0	0	1
Follicular thyroid carcinoma (FTC)	7	18	2
Minimally invasive FTC	5	16	2
Encapsulated angioinvasive FTC	2	1	0
Widely invasive FTC	0	1	0
Oncocytic carcinoma of the thyroid gland	2	16	2
Anaplastic thyroid carcinoma	0	0	1
Medullary thyroid carcinoma	1	0	1
Other neoplasms	2	1	0
Parathyroid adenoma	2	0	0
Malignant teratoma	0	1	0
Non-neoplastic	4	3	0
Necrosis/fibrosis	1	1	0
Inflammation	3	2	0

**Table A4:** Histopathological findings in Bethesda categories III, IV, and V thyroid nodules with molecular testing of FNA samples and surgical follow-up, stratified by molecular test result (n = 635).

Histopathological Diagnosis	*BRAF* (n = 51)	*HRAS* (n = 16)	*KRAS* (n = 5)	*NRAS* (n = 67)	*RET/* *PTC1* (n = 2)	*PAX8/* *PPARγ* (n = 5)	Neg (n = 439)	IC (n = 50)
Hyperplastic nodule		2	2	8			143	22
Follicular thyroid adenoma		11	2	43		2	215	16
NIFTP				7			9	1
Papillary thyroid carcinoma (PTC)	51			3	2	1	27	9
Infiltrative follicular variant of PTC	14			2	0	1	13	2
Oncocytic PTC	1			0	0	0	1	0
Classic PTC	32			0	2	0	11	7
Encapsulated classic PTC	3			1	0	0	2	0
Tall cell PTC	1			0	0	0	0	0
Follicular thyroid carcinoma (FTC)		2		6		2	15	1
Minimally invasive FTC		1		5		2	13	1
Encapsulated angioinvasive FTC		1		1		0	1	0
Widely invasive FTC		0		0		0	1	0
Oncocytic carcinoma of the thyroid gland							20	
Anaplastic thyroid carcinoma							1	
Medullary thyroid carcinoma		1					1	
Other neoplasms							2	1
Parathyroid adenoma							1	1
Malignant teratoma							1	0
Non-neoplastic			1				6	
Necrosis/fibrosis			1				1	
Inflammation			0				5	

One minimally invasive FTC with the detection of both an *HRAS* point mutation and a *PAX8/PPARγ* fusion was not included in this table. Neg, negative; IC, inconclusive.

**Table A5:** Histopathological findings in Bethesda category VI thyroid nodules with surgical follow-up, stratified by molecular test result (n = 61).

Histopathological Diagnosis	*BRAF* (n = 50)	*RET/PTC1* (n = 2)	*RET/PTC3* (n = 1)	Neg (n = 5)	IC (n = 3)
Papillary thyroid carcinoma (PTC)	50	2	1	4	2
Infiltrative follicular variant of PTC	6	0	0	3	0
Oncocytic PTC	0	0	0	0	0
Classic PTC	37	2	1	1	2
Encapsulated classic PTC	3	0	0	0	0
Tall cell PTC	4	0	0	0	0
Anaplastic thyroid carcinoma				1	
Medullary thyroid carcinoma					1

**Table A6:** Gene alteration-associated ROM in Bethesda categories III, IV, and V thyroid nodules with molecular testing of FNA samples and surgical follow-up, stratified by Bethesda category.

Molecular Test Result	Bethesda III (n = 213)	Bethesda IV (n = 329)	Bethesda V (n = 93)
*BRAF* V600E (n = 51)	100% (29.24–100%)	100% (29.24–100%)	100% (92.13–100%)
*HRAS* (n = 16)	14.29% (0.36–57.87%)	12.5% (0.32–52.65%)	100% (2.5–100%)
*KRAS* (n = 5)	0% (0–60.24%)	0% (0–97.5%)	-
*NRAS* (n = 67)	13.79% (3.89–31.66%)	9.68% (2.04–25.75%)	28.6% (3.67–70.96%)
*RET/PTC1* (n = 2)	-	-	100% (15.81–100%)
*PAX8/PPARγ* (n = 5)	0% (0–97.5%)	66.7% (9.43–99.16%)	100% (2.5–100%)
Negative (n = 439)	6.08% (2.81–11.23%)	12.98% (9.16–17.66%)	75.9% (56.46–89.70%)
Inconclusive (n = 50)	9.52% (1.17–30.38%)	4.76% (0.12–23.82%)	87.5% (47.35–99.68%)

One minimally invasive FTC with the detection of both an *HRAS* point mutation and a *PAX8/PPARγ* fusion was not included in this table.

**Table A7:** Surgical management of patients with Bethesda categories III, IV, or V thyroid nodules with molecular testing of FNA samples, stratified by molecular test result.

	Positive	Negative	Inconclusive
**Initial management**			
Hemithyroidectomy (HT)	111 (67.68%)	346 (60.17%)	38 (58.46%)
Total thyroidectomy (TT)	27 (16.46%)	54 (9.39%)	9 (13.85%)
Partial resection ^1^	4 (2.44%)	9 (1.57%)	1 (1.54%)
No surgery	22 (13.41%)	166 (28.87%)	17 (26.15%)
**Histology after HT**			
Malignant	47 (42.34%)	53 (15.32%)	6 (15.80%)
Benign	64 (57.66%)	293 (84.68%)	32 (84.21%)
**Histology after TT**			
Malignant	16 (59.26%)	6 (11.11%)	4 (44.44%)
Benign	11 (40.74%)	48 (88.89%)	5 (55.56%)
**Completion thyroidectomy after malignant histology in HT**			
Yes	25 (53.19%)	30 (56.60%)	4 (66.67%)
No	22 (46.81%)	23 (43.40%)	2 (33.33%)

Patients with more than one thyroid nodule were included in the “positive” group if there was a gene alteration detected in at least one of the nodules. ^1^ Includes isthmus resections and resections of remaining thyroid tissue after total thyroidectomy.
