# Supplementary material for: Performance of Seven-Gene Panel Testing for Risk Stratification of Thyroid Nodules with Indeterminate Cytology Results
Source: Int J Mol Sci. 2026 May 30;27(11):4990. doi: 10.3390/ijms27114990 (PMC13256701; doi:10.3390/ijms27114990)
Supplement: Supplementary file 1 [file ijms-27-04990-s001.zip › ijms-4257475-supplementary.pdf]

Table S1. Results of molecular testing of hyperplastic nodules with *RAS* mutations.

Patient ID	Molecular testing of FNA sample	Histological diagnosis after resection	Molecular testing of resection material
106	*NRAS* mutation	Hyperplastic nodule	*NRAS* p.Q61R (c.182A>G)
144a	*NRAS* mutation	Hyperplastic nodule	*NRAS* p.Q61R (c.182A>G)
333a	*NRAS* mutation	Hyperplastic nodule	inconclusive
355	*KRAS* mutation	Hyperplastic nodule	negative
377	*NRAS* mutation	Hyperplastic nodule	*NRAS* p.Q61R (c.182A>G)
444	*NRAS* mutation	Hyperplastic nodule	*NRAS* p.Q61R (c.182A>G)
574	*HRAS* mutation	Hyperplastic nodule	*HRAS* p.Q61R (c.182A>G)
680	*NRAS* mutation	Hyperplastic nodule	*NRAS* p.Q61R (c.182A>G)
796	*HRAS* mutation	Hyperplastic nodule	*HRAS* p.Q61R (c.182A>G)
800	*NRAS* mutation	Hyperplastic nodule	inconclusive
821	*KRAS* mutation	Hyperplastic nodule	*KRAS* p.G12D (c.35G>A)
854	*NRAS* mutation	Hyperplastic nodule	*NRAS* p.Q61R (c.182A>G)

Table S2. Correlation of core characteristics with ROM in Bethesda category III and IV thyroid nodules with molecular testing of FNA samples and surgical follow-up (n=495). Nodules with a *BRAF* V600E variant, a *RET/PTC1* fusion, *KRAS* point mutations, or an inconclusive molecular test result were excluded. Odds ratios were calculated with univariate logistic regression.

Characteristic	Odds	95% CI	*p*-value
Age, years	0.99	0.97–1.01	0.14
Female sex (yes/no)	0.91	0.49–1.70	0.78
Size on US[1], mm	1.03	1.01–1.04	0.001
Bethesda IV vs. III	1.93	1.02–3.65	0.042
HRAS point mutation	1.90	0.52–6.87	0.33
NRAS point mutation	1.07	0.46–2.48	0.88
PAX8/PPARγ fusion	12.64	2.06–77.37	0.006

[1] Information was missing from nine patients.

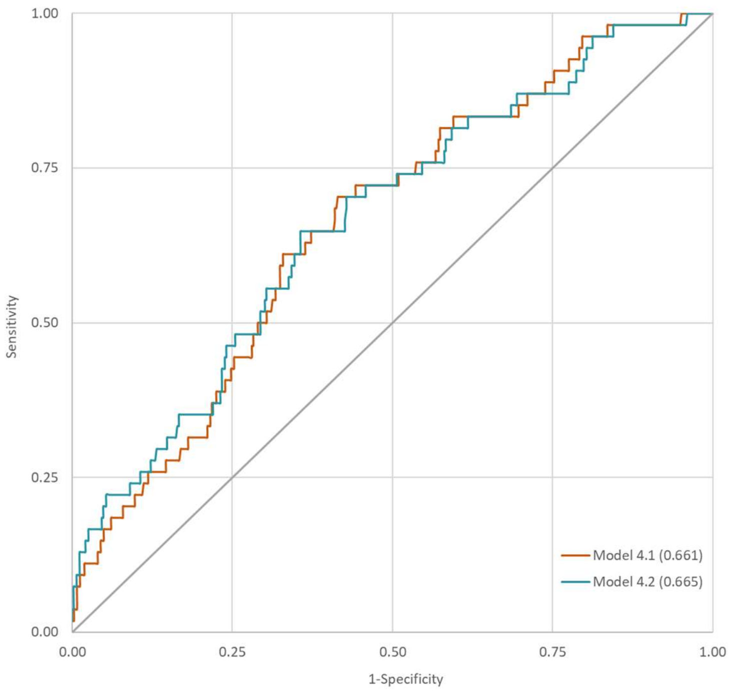

Figure S1. ROCAUC plot comparing multivariable regression models based on the core characteristics with or without the inclusion of genetic information in Bethesda III and IV thyroid nodules. Model 4.1 comprises the variables age, sex, size on US, and Bethesda category (IV vs. III). Model 4.2 is the same as Model 4.1 with added information about the presence of *HRAS* point mutations, *NRAS* point mutations, and *PAX8/PPARγ* fusions. The ROCAUC of the two models did not differ significantly from each other (Model 4.1, 0.661, 95% CI [0.587, 0.735] vs. Model 4.2, 0.665, 95% CI [0.589, 0.742], *p*=0.59).

Table S3. Correlation of extended characteristics with ROM in Bethesda category III and IV thyroid nodules with molecular testing of FNA samples and surgical follow-up (n=192). Nodules with a *BRAF* V600E variant, a *RET/PTC1* fusion, *KRAS* point mutations, or an inconclusive molecular test result were excluded. Odds ratios were calculated with univariate logistic regression.

Characteristic	Odds	95% CI	*p*-value
Age, years	1.00	0.96–1.03	0.77
Female sex (yes/no)	1.23	0.38–3.95	0.73
Height, mm	1.04	0.99–1.09	0.16
Length, mm	1.01	0.98–1.04	0.59
Width, mm	1.01	0.98–1.05	0.46
Taller than wide (yes/no)	1.33	0.28–6.32	0.72
Size on US, mm	1.01	0.98–1.04	0.53
Risk factors[1] (yes/no)	0.41	0.05–3.27	0.40
Known thyroid disease[2] (yes/no)	1.29	0.84–1.96	0.24
Symptoms[3] (yes/no)	0.99	0.33–2.94	0.98
Incidentaloma (yes/no)	0.67	0.23–1.99	0.47
**Margin**			
Sharp (vs. not)	0.34	0.09–1.36	0.13
Lobulated (vs. not)	4.47	1.07–18.78	0.041
**Texture**			
Solid (vs. not)	4.44	0.57–34.54	0.15
Partially cystic (vs. not)	0.26	0.03–2.02	0.20
**Echogenicity (categorical)**			0.050
Hyperechoic (vs. not)	1.76	0.20–15.55	0.61
Isoechoic (vs. not)	0.78	0.28–2.21	0.64
Hypoechoic (vs. not)	0.62	0.23–1.70	0.35
Marked hypoechoic (vs. not)	9.16	1.86–45.06	0.0064
**Focal changes (categorical)**			0.24
Macrocalcifications (vs. no changes)	10.87	0.65–182.2	0.097
Comet tail sign (vs. no changes)	1.14	0.14–9.59	0.90
Not classifiable (vs. no changes)	11.43	0.68–192.72	0.091
**EU-TIRADS, class**			0.083
4 (vs. 3)	0.52	0.15–1.85	0.31
5 (vs. 3)	2.34	0.72–7.61	0.16
**Bethesda, category**			
IV (vs. III)	1.56	0.53–4.63	0.42
**Mutation (yes/no)**	1.62	0.49–5.32	0.43
HRAS	2.67	0.28–25.37	0.39
NRAS	0.88	0.19–4.11	0.87
PAX8/PPARγ	10.88	0.65–182.21	0.097

[1]Risk factors include: family history of thyroid cancer in a first-degree relative, personal history of thyroid cancer, prior radiation therapy of the neck region, other radiation exposure, FDP-PET positivity of nodule, hereditary cancer syndromes such as MEN2. [2]Known thyroid diseases include: chronic autoimmune thyroiditis, Graves' disease, euthyroid struma, struma with hyperthyroidism, thyroid cancer. [3]Symptoms include: pain, difficulty swallowing, globus sensation, hoarseness. EU-TIRADS, European Thyroid Imaging Reporting and Database System.

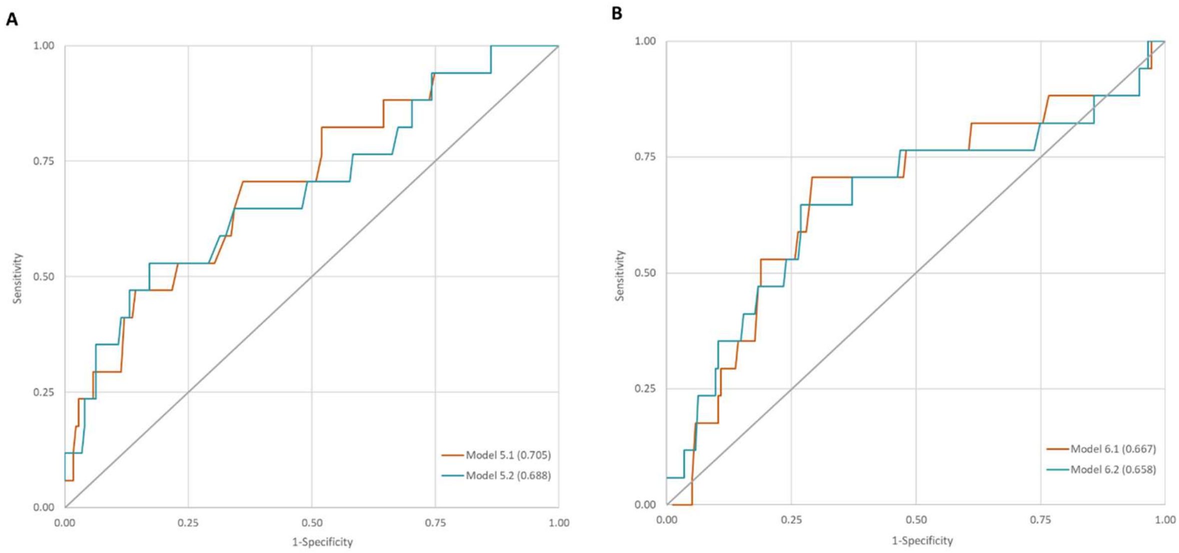

Figure S2. ROCAUC plot comparing multivariable regression models for Bethesda III and IV thyroid nodules based on the extended characteristics with or without the inclusion of genetic information. (**A**) Model 5.1 comprises the variables age, sex, margin (lobular vs. not), texture (solid vs. not), echogenicity (marked hypoechoic vs. not), and focal changes (present vs. not). Model 5.2 is the same as Model 5.1 with added information about the presence of *HRAS* point mutations, *NRAS* point mutations, and *PAX8/PPARγ* fusions. The ROCAUC of the two models did not differ significantly from each other (Model 5.1, 0.705, 95% CI [0.570, 0.841] vs. Model 5.2, 0.688, 95% CI [0.542, 0.835], p=0.46). (**B**) Model 6.1 comprises the variables age, sex, and EU-TIRADS (4 vs. 3, 5 vs. 3). Model 6.2 is the same as Model 6.1 with added information about the presence of *HRAS* point mutations, *NRAS* point mutations, and *PAX8/PPARγ* fusions. The ROCAUC of the two models did not differ significantly from each other (Model 6.1, 0.667, 95% CI [0.516, 0.819] vs. Model 6.2, 0.658, 95% CI [0.497, 0.820], *p*=0.70).

Table S4. Mean quantification cycle (Cq) values of the technical validation of the Thyroid Cancer Mutation Analysis Kit (*BRAF* V600E variant, *HRAS* mutations, *KRAS* mutations, *NRAS* mutations) with the AcroMetrix Oncology Hotspot Control at different dilutions.

Replicate number	Negative control	Positive control	Undiluted	1:10 dilution	1:100 dilution
1	0	30.26	30.81	33.17	37.22
2	0	29.86	30.84	33.02	36.76
3	0	29.95	30.34	32.74	36.63
4	0	30.78	31.18	33.75	37.19
5	0	30.55	31.09	33.05	36.71

Table S5. Mean Cq values of the technical validation of the Thyroid Cancer Fusion Gene Detection Kit (*RET/PTC1* fusions, *RET/PTC3* fusions, *PAX8/PPARγ* fusions) with the positive control included in the kit at different dilutions.

Replicate number	Negative control	Positive control	1:10 dilution	1:100 dilution
1	0	21.41	24.61	28.24
2	0	21.82	24.85	28.16
3	0	22.04	25.2	28.26

Table S6. Nodule characteristics of Bethesda category III, IV, and V thyroid nodules with surgical follow-up compared to those without surgical follow-up.

Nodule characteristic	With surgical follow-up (n=636)	Without surgical follow-up (n=213)
**Size on US** (in mm; median [interquartile range])	24 (17 – 34)	20 (15 – 27)
**EU-TIRADS***		
2	1 (0.35%)	2 (2.60%)
3	115 (40.49%)	30 (38.96%)
4	109 (38.38%)	29 (37.66%)
5	59 (20.77%)	16 (20.78%)
**Bethesda category**		
III	213 (33.49%)	157 (73.71%)
IV	330 (51.89%)	52 (24.41%)
V	93 (14.62%)	4 (1.88%)
**Molecular test result**		
Positive	147 (23.11%)	24 (8.45%)
Negative	439 (69.03%)	171 (80.28%)
Inconclusive	50 (7.86%)	18 (8.45%)
**Gene alteration**		
BRAF V600E	51 (8.02%)	3 (1.41%)
HRAS mutation	16 (2.52%)	3 (1.41%)
KRAS mutation	5 (0.79%)	3 (1.41%)
NRAS mutation	67 (10.53%)	15 (7.04%)
PAX8/PPARγ fusion	5 (0.79%	-
RET/PTC1 fusion	2 (0.31%)	-
HRAS mutation + *PAX8/PPARγ* fusion	1 (0.16%)	-
None	439 (69.03%)	171 (80.28%)
Inconclusive	50 (7.86%)	18 (8.45%)

*only available for a subgroup of patients (n=361)
